# Conservation—Oriented Analysis of *Apocynum venetum*’s Distribution in Response to Climate Change Based on MaxEnt Model

**DOI:** 10.3390/plants15060876

**Published:** 2026-03-12

**Authors:** Yong Chen, Jiali Cheng, Yuan Chen, Pengbin Dong, Liyang Wang, Hongwei Yang, Ru Chen, Juanli Wang

**Affiliations:** 1 Urumqi Comprehensive Experimental Station, Xinjiang Academy of Agricultural Sciences, Urumqi 830091, China; chenyong@xaas.ac.cn (Y.C.);; 2 College of Agronomy, Gansu Agricultural University, Lanzhou 730070, China; 3 College of Agronomy, Xinjiang Agricultural University, Urumqi 830052, China

**Keywords:** *Apocynum venetum* L., global climate change, shift prediction, potential distribution

## Abstract

In recent years, global climate change, combined with increased human activities, has led to habitat degradation and range shifts in rare medicinal plants, potentially affecting the quality of medicinal herbs. In this study, we assessed how key environmental variables shape the potential distribution of *Apocynum venetum* L. based on 281 wild occurrence records and nine environmental variables using the MaxEnt model. The results revealed that the mean temperature of the coldest quarter, solar radiation in June, and elevation are the most significant factors affecting the distribution of *A. venetum*, with optimal values ranging from −10 to 5 °C, 21,000 to 23,000 kJ m^−2^ day^−1^, and 200 to 1500 m, respectively. Ecological niche modeling indicated that highly suitable habitats are primarily located in Xinjiang, Gansu, Shaanxi, Shanxi, Henan, Hebei, Jiangsu, Shandong, and Inner Mongolia. However, future projections under climate change suggest an expansion of these suitable areas, shifting towards higher latitudes in the northwestern regions and high-altitude mountains. These findings provide a scientific basis for guiding the production and sustainable utilization of *A. venetum* resources.

## 1. Introduction

Global climate change has become a major challenge to the sustainable utilization of medicinal plants. Currently, the majority of medicinal plants supplied to the global market still primarily rely on wild harvesting [1]. Numerous studies have indicated that under future climate change scenarios, the suitable distribution range of many medicinal plants may shrink or undergo significant shifts [2,3]. Recent studies have found that under current and future climate scenarios, the habitat suitability centroid of tobacco (*Nicotiana tabacum* L.) will shift southeastward to varying degrees [4]. Under the three climate change scenarios, the suitable distribution area of *Leonurus japonicus* will shift toward higher latitudes [5]. Although some medicinal plants may sustain their populations by dispersing into newly suitable habitats, resource depletion could result in severe economic losses for local communities that rely on these plants [6]. Taking licorice (*Glycyrrhiza* spp.) as an example, its traditional distribution is in northern China. However, due to significant population decline and habitat destruction, China has shifted from being a primary producer to a major importer of this medicinal plant [7]. In recent years, the transition from reliance on unsustainable resource utilization to a sustainable development model has become a focal point for both academia and policymakers [8]. Within the context of medicinal plant conservation and sustainable utilization, commercial cultivation is considered a crucial strategy for protecting high-value medicinal plants while also promoting rural economic growth [3]. Therefore, accurately predicting changes in the suitable habitats of key medicinal plants and gaining deeper insights into their responses to climate change are of great significance for agricultural policy formulation and medicinal plant cultivation planning [9].

In studies exploring the impact of climate change on plant geographic distribution, the use of ecological niche models to predict species distribution has become a key trend in habitat suitability research. Ecological niche models, based on ecological principles, niche theory, and specific algorithms, utilize known plant distribution data and environmental variables to determine niche characteristics, thereby predicting suitable habitats for the species [10,11]. Based on different algorithmic rules and predictive objectives, species distribution models (SDMs) have evolved into diverse modeling approaches, including the Maximum Entropy Model (MaxEnt), the CLIMEX population growth model, the BIOCLIM model based on bioclimatic data, the Artificial Neural Network (ANN) model, the Ecological Niche Factor Analysis (ENFA) model, and the Genetic Algorithm for Ruleset Prediction (GARP) model [12]. Among these models, the MaxEnt model, proposed by Phillips in 2004, has demonstrated superior predictive performance. Based on the maximum entropy theory, the MaxEnt model is a density estimation and species distribution prediction tool characterized by short computation time, ease of operation, low sample size requirements, high predictive accuracy, and strong stability [13]. This model has been widely applied in delineating conservation areas for endangered species, analyzing the growth environments of medicinal plants, and predicting the potential distribution of invasive species [14,15]. Hu et al. [16] utilized this model to predict the suitable habitat distribution of the medicinal plant *Gastrodia elata* under future climate scenarios. Wang et al. [17] analyzed the key environmental factors affecting the growth of *Codonopsis radix* using the MaxEnt model. Yang et al. [18] employed this model to examine the environmental variables influencing the potential distribution of *Astragalus membranaceus* var. mongholicus and its suitable habitat, yielding valuable insights for species cultivation.

*Apocynum venetum* (commonly known as “Zeqi” in traditional Chinese medicine) has been highly valued for its medicinal properties since ancient times [19]. Its roots, stems, leaves, and flowers are all used in herbal medicine, demonstrating significant efficacy in treating cardiovascular diseases, hypertension, and bronchitis [20]. It has also been officially included in the Chinese Pharmacopoeia and the list of medicinal and edible homologous substances. The primary bioactive compounds of *A. venetum* are flavonoids, such as quercetin, rutin, and cardiac glycosides, which exhibit antihypertensive, antioxidant, and antidepressant pharmacological effects [21,22]. Additionally, the roots of *A. venetum* can penetrate 0.5–3.0 m into the soil, contributing to its exceptional stress resistance. It is highly tolerant to drought, salinity, freezing, high temperatures, and wind erosion. With a perennial root system capable of surviving for over 30 years, *A. venetum* demonstrates remarkable adaptability and can be cultivated in saline-alkali soils to mitigate land degradation [23]. However, due to climate change, environmental degradation, and excessive harvesting, the distribution range of *A. venetum* has declined sharply [24,25]. Current research on *A. venetum* primarily focuses on its pharmacological properties, chemical composition, antibacterial fiber properties, and genetic diversity. However, studies investigating the effects of environmental factors on its distribution and growth using species distribution models (SDMs) in combination with high-resolution general circulation models (GCMs) remain limited [26,27,28,29].

**Figure 1:**
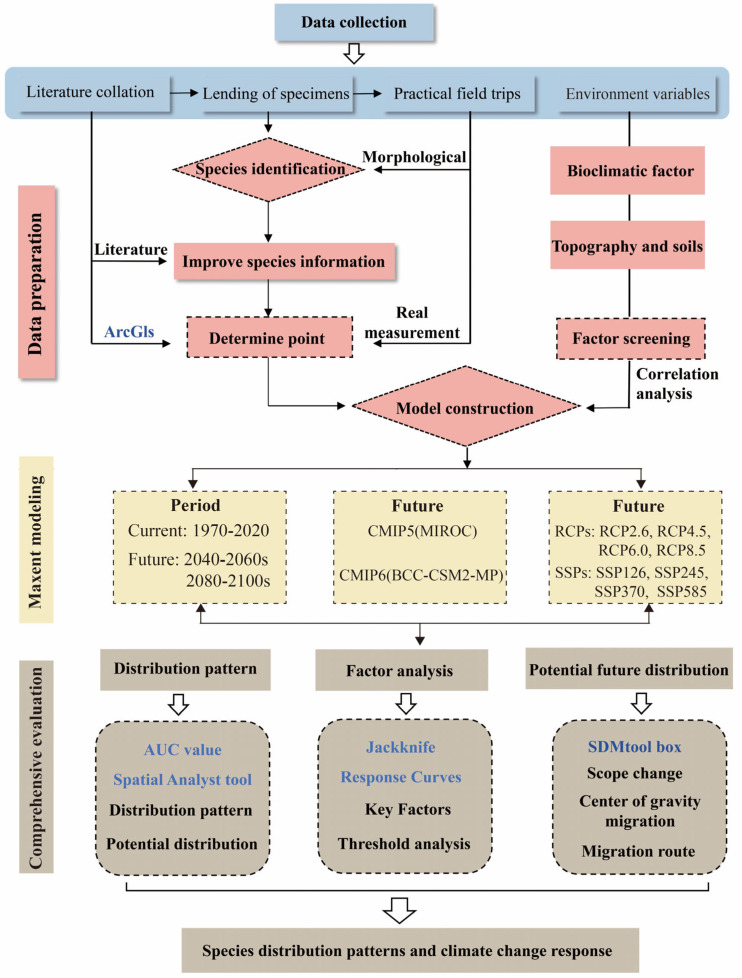
Schematic illustration of the assessment strategy for the impact of ecological factors on the ecological suitability and quality of *A. venetum*.

This study uses the MaxEnt model to assess how key environmental variables influence the current distribution of *A. venetum* and to predict potential changes in suitable habitats under future climate scenarios. The findings will provide a theoretical basis for conservation efforts and artificial cultivation, contributing to a better understanding of the impact of climate change on plant range shifts in arid regions and supporting sustainable ecological development. Figure 1 shows a schematic representation of the proposed methodology.

## 2. Materials and Methods

### 2.1. Sources of Data on the Geographical Distribution of A. venetum

**Figure 2:**
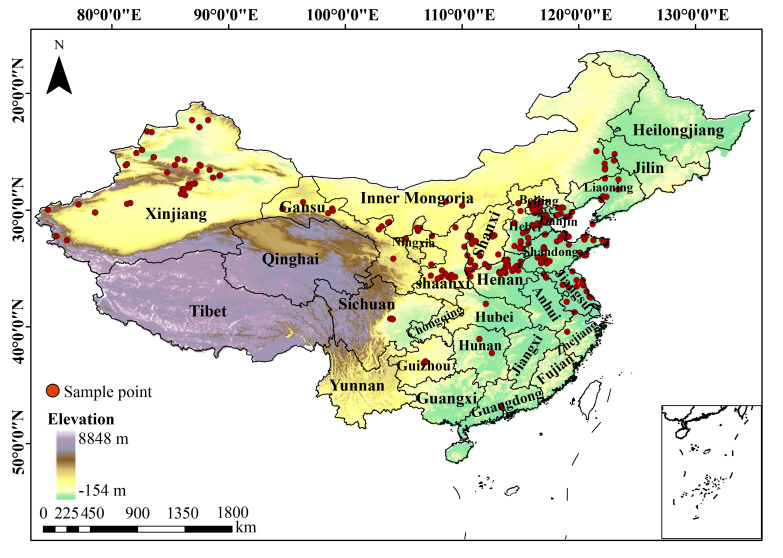
Geographical distribution of *A. venetum* in China. Note: red points represent species distribution records.

The geographical distribution data of *A. venetum* were collected through three primary methods: (1) field surveys were conducted in China during the summer of 2024, focusing on historical distribution areas of *A. venetum* and recording latitude and longitude coordinates via GPS; (2) database searches, including resources such as the Chinese Herbarium (CVH, https://www.cvh.ac.cn/) and the Global Biodiversity Information Facility (GBIF, https://www.gbif.org/); and (3) literature reviews of journal articles related to *A. venetum* from the past 50 years. For records with only administrative district information, geographical coordinates were retrieved using Google Maps (web version, accessed in 2025), with latitude and longitude data rounded to five decimal places. To ensure the accuracy of the species distribution data and avoid potential biases, we manually excluded records from cultivated sites, such as botanical gardens, greenhouses, and experimental plantations, as these locations may not accurately reflect the natural distribution of *A. venetum*. Additionally, incorrect species distribution records and points with vague location details (i.e., only at the county level) were removed. To reduce potential spatial clustering that could lead to sampling bias, we employed a spatial thinning method, retaining only one data point per 10 km radius. This approach minimized spatial autocorrelation in the distribution data and ensured a more representative sampling across diverse environmental conditions. After filtering, a total of 281 distribution points were documented (Figure 2).

### 2.2. Environmental Data Collection and Screening

In this study, a total of 39 environmental variables were incorporated, including 19 bioclimatic variables, seven soil variables, elevation, and solar radiation (Table S2). The bioclimatic variables, solar radiation, and elevation data were obtained from the WorldClim database (https://www.worldclim.org/) at a spatial resolution of 2.5 arc-minutes, spanning the period from 1970 to 2000 [30]. Soil data were obtained from the HWSD database (http://www.fao.org/soils-portal/, accessed on 11 January 2025) [31].

For future projections, Shared Socioeconomic Pathways (SSPs) and Representative Concentration Pathways (RCPs) were used to drive the models. Future temperature and precipitation projections were derived from three high-resolution general circulation models (MIROC5, BCC-CSM2-MR, and BCC-CSM1.1), with climate change data for MIROC5 and BCC-CSM1.1 obtained from the Fifth Assessment Report (AR5) of the Intergovernmental Panel on Climate Change (IPCC) [32,33]. These models simulated four distinct RCPs: RCP2.6, RCP4.5, RCP6.0, and RCP8.5. In addition, BCC-CSM2-MR (National Climate Center, Beijing) from the Coupled Model Intercomparison Project Phase 6 (CMIP6) was used to project climate conditions in China under four SSP scenarios (SSP126, SSP245, SSP370, and SSP585). The potential distribution of *A. venetum* was projected for the 2050s and 2090s. To maintain consistency across temporal models, soil and topographic variables were kept constant for future predictions [34,35]. Therefore, the same soil and topographic variables were used in both current and future distribution analyses.

**Figure 3:**
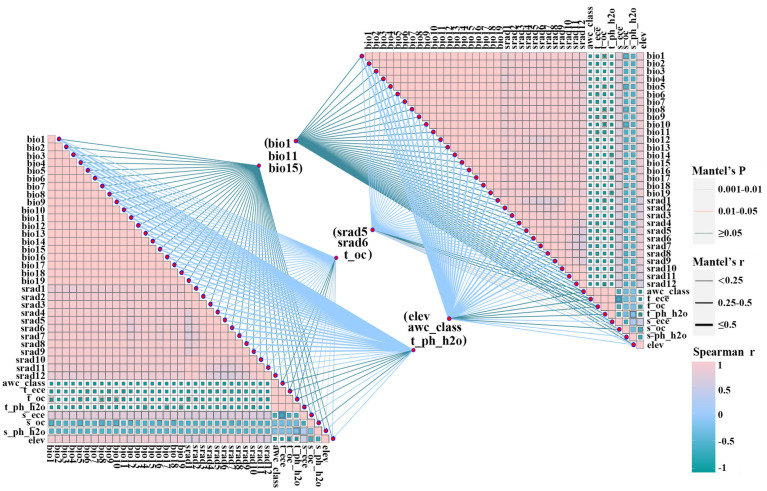
Correlation analysis of environmental variables. Blue represents positive correlations and yellow represents negative correlations.

To enhance prediction accuracy and avoid overfitting among environmental factors, the MaxEnt model was initially employed to simulate *A. venetum* distributions using 39 environmental climate variables, excluding those bioclimatic factors with zero contribution [36]. Specifically, environmental values at occurrence points were extracted in ArcGIS 10.2, and Spearman’s rank correlation coefficients (r) were calculated to identify highly correlated variable pairs (|r| > 0.8) [37]. For each highly correlated pair, only the variable with higher contribution in the initial MaxEnt model and stronger ecological relevance was retained, ensuring that the final model included variables that were both informative and ecologically interpretable (Figure 3) [38]. In addition, Mantel tests and correlation visualization were conducted using ChiPlot (https://www.chiplot.com/). Environmental distance matrices and species distribution distance matrices were generated based on standardized predictor variables and presence/background points, and uploaded to ChiPlot for Mantel testing with 999 permutations to obtain the Mantel correlation coefficient and significance level. ChiPlot was also used to generate composite plots integrating Mantel network connections with Spearman correlation matrices for clear visualization of variable relationships.

Ultimately, nine environmental variables were chosen for the MaxEnt model, including one terrain variables (elevation), three climate variables bio2: mean diurnal range (mean of monthly), bio11: mean temperature of coldest quarter, bio15: precipitation seasonality (coefficient of variation), solar radiation, and three soil factors (Awc_class, T_oc, and T_ph_h2o) (Table 1).

### 2.3. MaxEnt Model Operation and Evaluation

In constructing distribution models, the system provides a set of default parameters, but these parameters are influenced by the test data. The default parameter settings can increase the model’s sensitivity to the test data, potentially leading to overfitting and compromising the accuracy of the model’s predictions. In this study, the Kuenm package in R (version 1.1.7) was used to optimize key parameters in the MaxEnt model, specifically the feature class (FC) and the regularization multiplier (RM) [39]. The RM value was set to range from 0.1 to 4, increasing in increments of 0.1. The five FCs (linear-L, quadratic-Q, product-P, threshold-T, and hinge-H) were permuted into 31 FC combinations. The fit and complexity of these parameter combinations were assessed using the Akaike Information Criterion correction (AICc) [39,40]. To assess the model’s ability to discriminate suitable from unsuitable conditions, 10,000 background points were randomly sampled across the study area to represent the range of available environmental conditions, following standard MaxEnt procedures [13,41]. The area under the receiver operating characteristic (ROC) curve (AUC) and true skill statistic (TSS) [42] was used to quantify the model’s discriminatory performance. These background points do not indicate true species absence but serve as reference locations to contrast the environmental characteristics of known occurrence points. Based on the optimization results, we selected a model with an omission rate of less than 0.05 and a small-sample corrected delta Akaike Information Criterion (ΔAICc) of ≤2 [43].

### 2.4. Areas of Suitability

To classify and visualize the suitable habitats, and to create a potential distribution map of *A. venetum*, the ASCII outputs from the MaxEnt model were imported into ArcGIS 10.6 for spatial analysis and visualization. The continuous logistic outputs (ranging from 0 to 1) were converted into binary suitable and unsuitable habitats using the maximum training sensitivity plus specificity threshold (MTSPS) provided by MaxEnt. This threshold maximizes the sum of sensitivity and specificity and is equivalent to maximizing the True Skill Statistic (TSS), thereby balancing omission and commission errors [44,45]. The use of this threshold has been widely recommended for generating presence–absence maps in species distribution modeling studies. Areas with suitability values greater than the MTSPS were considered suitable habitats, whereas those below the threshold were classified as unsuitable habitats. To further characterize habitat suitability gradients, only the suitable areas (values > MTSPS) were subdivided into low, moderate, and high suitability classes. The 25th percentile (Q1) and 75th percentile (Q3) of the predicted suitability values within the suitable range were calculated, and habitats were classified as follows: low suitability (MTSPS–Q1), moderate suitability (Q1–Q3), and high suitability (>Q3). This data-driven classification approach avoids arbitrary threshold selection and better reflects the statistical distribution of model predictions [46].

### 2.5. Analysis of Centroid Shift in Suitable Distribution Areas

The species distribution modeling (SDM) tool is commonly used to calculate the centroid points of a species and assess its shift distances based on latitude and longitude coordinates [47]. In this study, we used the SDM_Toolbox_v2.4 package (Carbondale, IL, USA) to calculate the centroid position of the suitable habitat for *A. venetum*. Comparisons of centroid shifts under various climate scenarios were made against the current baseline for the 2050s and 2090s. To achieve this, we converted the predicted suitable habitat data from ASC format to binary TIFF files. Suitable habitats were defined as areas with a species suitability probability of *p* ≥ 0.5, while areas with *p* < 0.5 were considered non-suitable. We then used the centroid change (line) tool in the SDM_Toolbox_v2.4 package to select the centroid geometry type to derive the coordinates of the centroid position in suitable habitat for *A. venetum* under specific climate scenarios and periods. The displacement distances of the centroids across scenarios were subsequently calculated.

## 3. Results

### 3.1. MaxEnt Model Evaluation

The average training AUC and average TSS from repeated runs were 0.966 and 0.835, respectively (Table S1), indicating that the reconstructed model exhibited high predictive performance and reliability. In species distribution modeling, an AUC value greater than 0.9 generally reflects excellent model discrimination ability, while high TSS values indicate strong agreement between predicted and observed distributions. These results suggest that the model can effectively distinguish suitable from unsuitable habitats and provides robust predictions of the potential suitable habitats of *A. venetum*. Therefore, the model can be considered reliable for subsequent analyses of habitat suitability and distribution patterns.

### 3.2. Analysis of Environmental Factor Importance

**Figure 4:**
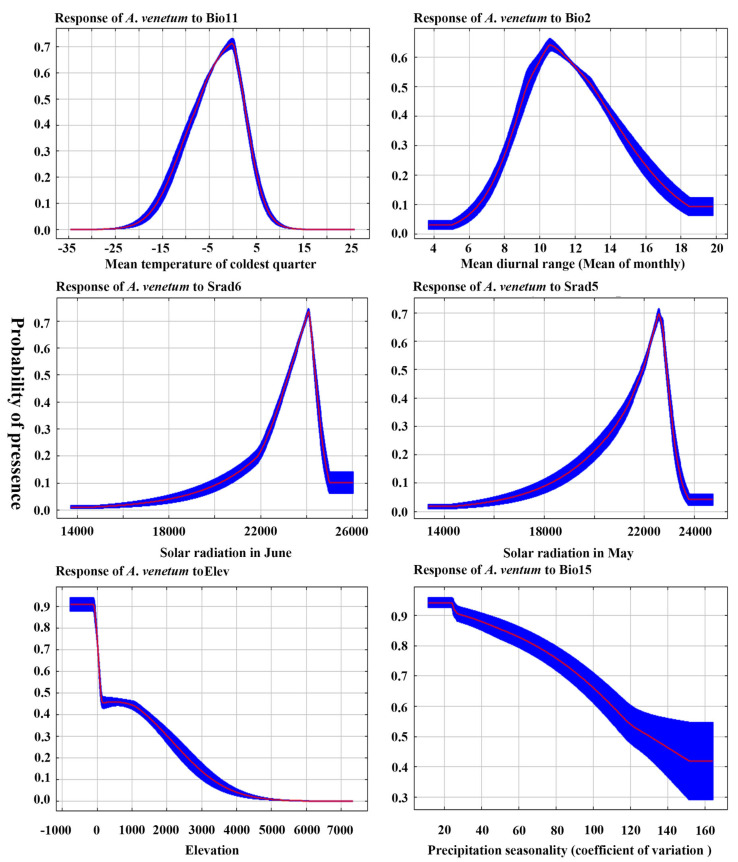
Marginal response curves of the current existence probability of *A. venetum* to the bioclimatic variables.

The results of the jackknife test revealed that the contribution of each climatic factor. As shown in Table S3, the key variables influencing the distribution of *A. venetum* under the current climate scenario include Srad6 (solar radiation in June), Bio11 (mean temperature of coldest quarter), Elev (Elevation), Awc_class (AWC range), Srad5 (Solar radiation in May), and T_ph_h2o (Topsoil pH (H_2_O)). Among these variables, Srad6 contributes the most to the potential distribution of *A. venetum*, accounting for approximately 38.5%, followed by Bio11 (18.1%), Elev (11.5%), Awc_class (8.5%), Srad5 (6.2%), and Bio15 (5.5%). Based on the marginal response curves of the primary ecological factors for *A. venetum*, the optimal range for Bio11 is −10 to 5 °C, the optimal range for Srad6 is 23,000 to 24,000 kJ·m^−2^·day^−1^, the optimal range for Srad5 is 21,000 to 23,800 kJ·m^−2^·day^−1^, and the optimal range for Elev is 200 to 1500 m (Figure 4).

### 3.3. Current Potential Distribution Estimates

**Figure 5:**
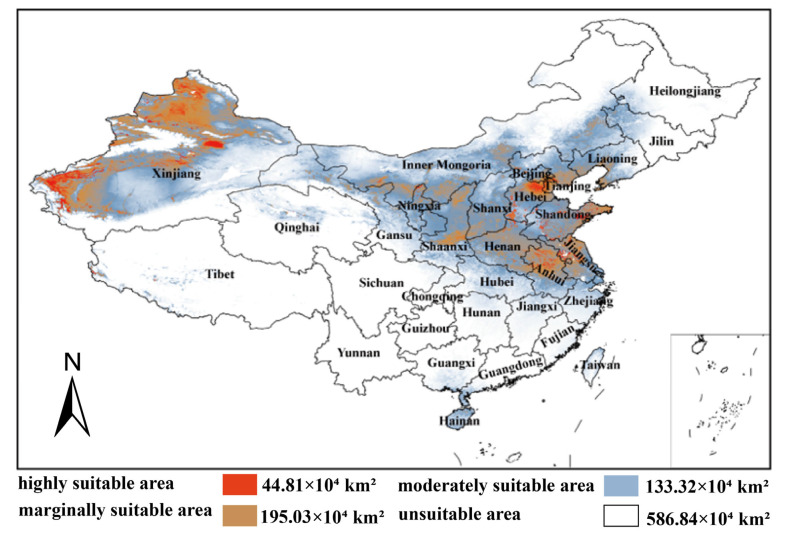
Predicted suitable habitats of *A. venetum* in China based on MaxEnt modeling. Colors indicate suitability categories: highly suitable (44.81 × 10^4^ km^2^), moderately suitable (133.32 × 10^4^ km^2^), marginally suitable (195.03 × 10^4^ km^2^). Total suitable area is 373.16 × 10^4^ km^2^ (~38.87% of China’s territory).

Model results (Figure 5) showed that the distribution of the species was mainly concentrated in the north of China. The ecological niche modeling indicates that the highly suitable area for *A. venetum* is approximately 44.81 × 10^4^ km^2^, the moderately suitable area is 133.32 × 10^4^ km^2^, and the marginally suitable area is 195.03 × 10^4^ km^2^, with a total suitable habitat area of approximately 374.03 × 10^4^ km^2^, accounting for 38.87% of China’s territory. Although this species is absent in some forecasted regions, the primary distribution areas predicted by the model align with current distribution records. The highly suitable areas for *A. venetum* are mainly distributed in Xinjiang, Gansu, Shaanxi, Shanxi, Henan, Hebei, Jiangsu, Shandong and Inner Mongolia (Figure 5).

### 3.4. Suitable Distribution Under Future Climate Scenarios

**Figure 6:**
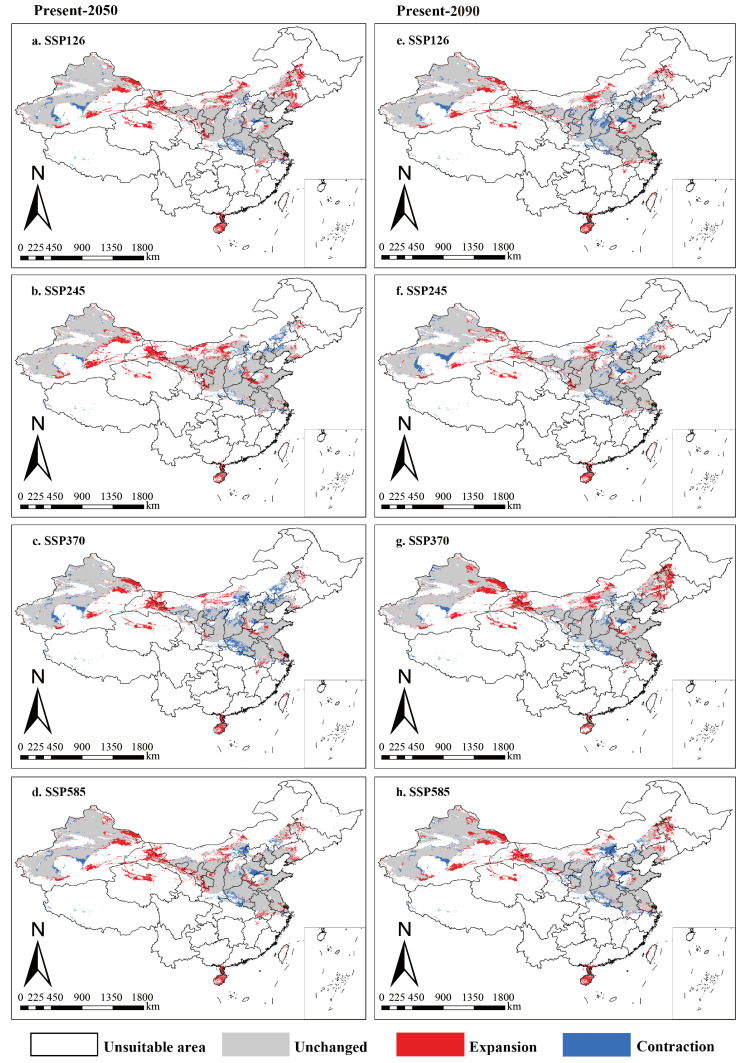
Spatial changes in *A. venetum* in China under emission scenarios of the 2050s and 2090s. White, Gray, Red and Blue areas represent not suitable, unchanged suitable, expansion suitable, and contraction suitable areas, respectively.

In the 2050s and 2090s, under four future climate scenarios, the predicted suitable distribution areas of *A. venetum* exhibit distinct patterns (Figure 6 and Figure S1, and Table 2). The BCC-CSM2-MR model of the Shared Socioeconomic Pathways projects suitable areas to expand mainly into northern Xinjiang, the Hexi Corridor, central Qinghai, and northern Inner Mongolia, while parts of northern Shanxi, eastern Henan, and central Hebei contract (Figure 6). In the analysis using RCP scenarios with the MIROC5 model, suitable habitats for *A. venetum* are projected to expand toward central and northern Xinjiang, the Hexi Corridor, central Qinghai, central Inner Mongolia, western Heilongjiang, western Jilin, and northern Shanxi (Figure S1). Meanwhile, suitable areas in central northern Xinjiang, northern Hebei, and eastern Inner Mongolia are anticipated to shrink, with parts of Qinghai also exhibiting a shrinking trend towards the southeast (Figure S1). Regarding changes in suitable area size, most future climate scenarios show an overall increasing trend in suitable regions. The statistical results of the BCC-CSM2-MR and MIROC models show that the suitable area increased under the four different emission scenarios (Figure 6 and Figure S1, and Table 2).

### 3.5. The Shift Trends of the Geometric Center of Suitable Habitat

**Figure 7:**
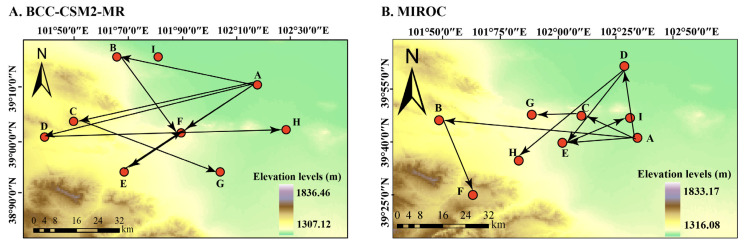
Shift in the center of suitable habitat for A. venetum under the future climate scenarios based on different climate models. (**A**) BCC-CSM2-MR model; (**B**) MIROC model. Arrows indicate the migration routes and directions of the suitable habitat distribution center under different climate scenarios. The letters represent different scenarios/periods: A, current; B, RCP2.6–2050s; C, RCP4.5–2050s; D, RCP6.0–2050s; E, RCP8.5–2050s; F, RCP2.6–2090s; G, RCP4.5–2090s; H, RCP6.0–2090s; and I, RCP8.5–2090s. Background colors represent elevation levels.

Currently, the geometric center of the potential suitable habitat for *A. venetum* is located in Hongshagang Town, Minqin County, Wuwei City, Gansu Province, China (102.28546143° E, 39.18011074° N). Future projections indicate that, relative to the current distribution, the centroid of suitable habitat in China exhibits an overall northwestward shift. Across all RCP scenarios, the migration trajectories consistently show a predominant northwestward movement, suggesting that under climate change, the suitable habitat tends to shift toward higher latitudes or transitional arid regions. However, variations among RCP scenarios are evident. Some scenarios display more pronounced shifts during the 2050s, whereas directional adjustments or reduced displacement occur by the 2090s (Figure 7). These patterns indicate that centroid migration is not strictly linear, but rather reflects a dynamic reorganization of suitable habitat under different climate change pathways. Moreover, based on the MIROC model, the shift distances under the RCP2.6, RCP4.5, RCP6.0, and RCP8.5 scenarios are estimated to be 54,700 m, 14,159 m, 19,705 m, and 19,373 m, respectively, for the 2050s (Figure S2A); for the 2090s, under the same scenarios, the centers of suitable areas are expected to shift northwest with distances of 49,676 m, 23,915 m, 34,150 m, and 60,266 m, respectively (Figure S2B).

## 4. Discussion

### 4.1. Model Uncertainty and Limitations

In this study, topographic and soil variables were assumed to remain constant throughout the 21st century, whereas only climatic variables were projected under future scenarios. Topographic variables (e.g., elevation, slope, and aspect) are generally considered stable over ecological time scales and are therefore commonly treated as static predictors in species distribution models (SDMs) [48,49]. However, soil properties may change over time as a consequence of climate-driven processes, vegetation shifts, erosion, and land-use change. Because high-resolution, spatially explicit projections of future soil conditions are currently unavailable at regional and global scales, many SDM studies assume static edaphic variables when forecasting distributions under climate change [50]. This assumption inevitably introduces uncertainty into model outputs. Substantial shifts in soil properties under future climate regimes could lead to regional overestimation or underestimation of suitable habitat areas. More broadly, climate-driven SDMs primarily reflect potential climatic suitability rather than fully realized ecological suitability, as they do not explicitly incorporate dispersal limitations, biotic interactions, or dynamic soil processes [51,52]. Consequently, our projections should be interpreted as indicative trends in climate-driven habitat suitability rather than precise forecasts of realized species distributions.

In addition, human activities, land-use change, and biotic interactions—none of which were explicitly included in the present model-may further influence projected distributions. Anthropogenic activities such as irrigation, cultivation expansion, grazing, and infrastructure development can substantially modify local habitat conditions. In arid and semi-arid regions, artificial irrigation may buffer climatic stress and create microhabitats not fully captured by climate-based predictors, potentially resulting in underestimation of suitable areas in intensively managed landscapes. Conversely, land degradation, urbanization, and agricultural conversion may reduce habitat availability within climatically suitable zones, leading to overestimation of actual distributions [53]. Land-use change can also alter vegetation structure, soil properties, and hydrological processes independently of macroclimatic conditions. Because our model is primarily based on climatic, topographic, and soil variables, it characterizes the species’ potential ecological niche rather than its realized distribution under strong anthropogenic constraints [54].

Biotic interactions-including competition, facilitation, herbivory, and pollinator availability-may further restrict or shift the realized niche. For instance, competitive exclusion by drought-adapted C4 species in arid or high-elevation environments could limit establishment even where climatic conditions appear suitable. Conversely, positive plant–soil feedbacks or mutualistic interactions may enhance persistence beyond what abiotic predictors alone would suggest. Increasing evidence indicates that incorporating biotic interactions can substantially alter projected species distributions [55,56].

We acknowledge that integrating land-use variables, anthropogenic disturbance indices, and biotic interaction data would enhance the ecological realism of SDMs. In the present study, land-use–related datasets (e.g., Land Use Harmonization products) and explicit human-impact metrics were not incorporated due to uncertainties in future land-use projections and inconsistencies in spatial resolution across large-scale datasets. Nevertheless, integrating these factors would improve model realism and strengthen projections of species responses under future climate scenarios. Future research should therefore incorporate dynamic soil datasets, high-resolution land-use change scenarios, and anthropogenic impact indicators to better represent ecological processes and human pressures. Finally, uncertainty in SDMs arises not only from predictor selection but also from variability among climate models and emission pathways. Integrating multiple sources of uncertainty, including climate model variability, scenario selection, and predictor configuration, along with mechanistic or hybrid modeling approaches, would provide more robust and ecologically realistic assessments of species responses to climate change [57].

### 4.2. Influence of Environmental Factors on Habitats of Species

The distribution and ecological adaptability of *A. venetum* are closely linked to specific climate and soil factors, which directly regulate its physiological thresholds and habitat suitability. Our MaxEnt model analysis identified solar radiation, mean temperature of the coldest quarter (Bio11), and elevation as the dominant drivers of its current distribution. These factors collectively reflect *A. venetum*’s adaptation to warm, humid monsoon climates with seasonal aridity, while also highlighting its vulnerability to climatic extremes [58]

Solar radiation was the most critical factor, contributing 42.3% to the model’s predictive power. High solar radiation in June (23,000–24,000 kJ·m^−2^·day^−1^) enhances photosynthetic efficiency, promotes the accumulation of flavonoids and phenolic compounds that underpin its medicinal quality, particularly quercetin, and mitigates adverse effects of osmotic stress on seed germination under high temperatures [59,60]. However, radiation exceeding this range, observed in parts of northwestern China due to reduced cloud cover, induces oxidative stress, disrupting the balance between photosynthesis and antioxidant defense systems, which explains why *A. venetum*’s optimal habitats are retreating from low-eastern plains to moderately irradiated regions [61].

The mean temperature of the coldest quarter (Bio11: −10 to 5 °C) further constrains its distribution. Winter temperatures above 5 °C reduce vernalization efficacy, impairing bud dormancy release and flowering synchrony, while temperatures below −10 °C may cause xylem embolism due to the species’ narrow-vessel hydraulic architecture, limiting expansion into colder regions [62,63]. Bio11 thus constrains winter dormancy and germination rhythms; values above 5 °C reduce vernalization efficiency, whereas values below −10 °C can lead to xylem embolism, restricting the species’ distribution [64].

Elevational gradients (400–2800 m) mediate interactions between temperature and moisture availability. At lower elevations (<400 m), high evapotranspiration and saline soils restrict water availability, while at higher elevations (>2800 m), reduced CO_2_ partial pressure and prolonged frost periods suppress carbon assimilation and favor C_4_ competitors [65,66]. The species’ dominance in mid-elevation zones (900–1500 m) aligns with its ability to balance hydraulic safety and growth under moderate aridity. Elevation mediates temperature–moisture interactions, influencing soil water availability and carbon assimilation efficiency [67].

Soil variables (Awc_class, T_oc, T_ph_h2o) reflect water retention capacity, organic matter supply, and nutrient availability, which are critical for plant growth and habitat suitability [68,69]. Climate-driven soil degradation, including salinization and organic matter loss, amplifies these biotic thresholds. Although *A. venetum* exhibits high salt tolerance (Na^+^/K^+^ homeostasis up to 200 mM NaCl), synergistic stress from drought and salinity reduces seedling establishment by 60–75% in desert fringe areas [70].

The nine environmental variables included in the model (bio2, Bio11, Bio15, solar radiation, elevation, Awc_class, T_oc, T_ph_h_2_o) were selected using an integrated approach combining correlation analysis (|r| < 0.8), variable contribution assessment, and ecological interpretation, rather than relying solely on statistical thresholds. Temperature, precipitation, energy supply, topographic gradients, and soil properties collectively capture multiple ecological dimensions affecting species distribution, including thermal stress, water availability, energy input, and soil resource constraints [71,72].

Our projections suggest that targeted cultivation in northwestern China could exploit *A. venetum*’s ecological plasticity: its deep root system stabilizes sandy soils, while leaf litter deposition increases soil organic carbon by 1.2–1.8% annually in trial plots. However, microclimate management, such as windbreak nets to reduce radiation-induced photoinhibition, is recommended to align cultivation zones with the species’ evolving climatic niche.

### 4.3. Response of Spatial Distribution Pattern of A. venetum to Climate Change

At present, the potential distribution areas of *A. venetum* are primarily concentrated in Xinjiang, Gansu, Shaanxi, Shanxi, Henan, Hebei, Jiangsu, Shandong and Inner Mongolia (Figure 5). The predicted distribution aligns well with the recorded distribution of *A. venetum* in China as documented in the Flora of China. Compared to the current distribution, by the future (2050s and 2090s), the distribution of *A. venetum* is expected to increase in central and northern Xinjiang, the Hexi Corridor, central Qinghai, central Inner Mongolia, western Heilongjiang, western Jilin, and northern Shanxi compared to the current distribution (Figure 6 and Figure S1). Under a future scenario of significant warming of the climate, the trend of expanding the area of suitable areas and significant changes in spatial patterns is not only seen in the subjects of this study, but also reflected in other medicinal plants, such as *Hypericum perforatum* Linn., *Eremochloa ophiuroides* (Munro) Hack., and *Choerospondias axillaris* (Roxb.) B.L.Burtt & A.W.Hill [73,74,75]. With an expected average increase in global surface temperature of 1.4–5.8 °C by the end of the century and a further increase in the frequency and intensity of extreme precipitation events, about half of the world’s plant species are projected to be at risk, potentially leading to changes in 49% of terrestrial plant communities and 37% of terrestrial biomes [76,77]. However, the suitable distribution of medicinal plants may be influenced by biological, geological, or other disturbance factors, in addition to the effects of climate change [78]. *A. venetum* has the efficacy of strengthening the heart, anti-inflammation, relieving cough and asthma, and lowering blood lipids [79,80]. It is one of the major Chinese medicinal materials with the most development potential [81]. Here, two plausible reasons may explain why the suitable distribution areas of the *A. venetum* tend to expand in the future. Firstly, climate warming has a positive impact on the expansion of thermophilic plants, causing the expansion of thermophilic plants and the retreat of psychrophilic plants to the Antarctic and Arctic [82,83]. Secondly, precipitation is the biggest influencing factor; against the backdrop of global warming, China’s precipitation will continue to increase [84].

In the 2050s, under the SSP126, SSP245, SSP370, and SSP585 climate scenarios, the centroid of the overall suitable habitat for *A. venetum* will shift northwest to Hongshagang Town, Minqin County, Wuwei City, Gansu Province, with shift distances of 35,214 m, 49,988 m, 56,833 m, and 40,437 m respectively (Figure 7A). For the 2050s, under the RCP2.6, RCP4.5, RCP6.0, and RCP8.5 climate scenarios, these shift distances are estimated at 54,700 m, 14,159 m, 19,705 m, and 19,373 m, respectively (Figure 7B). In addition, in the emission scenarios for the 2050s and 2090s, the shift route of the centroid tends to shift toward higher latitude areas. In the context of future global warming, the suitable habitat for *A. venetum* will overall shift towards higher latitude areas, and the shift distance will increase with the intensity of the representative concentration pathways.

The non-linear differences between the 2050s and 2090s indicate that the response of *A. venetum* to climate change is stage-dependent rather than temporally linear. Climate-driven biodiversity redistribution is often characterized by complex spatial reorganization rather than simple linear expansion or contraction [85]. When climatic conditions shift toward the species’ optimal niche, suitable areas may increase; however, further climatic changes exceeding tolerance limits may lead to redistribution or contraction. Differences between climate model projections further highlight the influence of model uncertainty. Recent studies emphasize that SDM outcomes are strongly dependent on scenario selection and model structure [86]. Moreover, varying emission pathways under the CMIP6 SSP framework can produce divergent long-term projections [87]. Therefore, the observed non-linear temporal patterns likely reflect the combined effects of scenario intensity and climate model uncertainty.

However, it is important to note that the shift analysis presented in Figure 7 is based solely on shifts in the spatial centroids of predicted suitable areas, without accounting for ecological or geographical constraints such as mountain ranges, deserts, or land-use patterns. As a result, the directions and distances of centroid shifts do not represent actual species movement pathways or biological feasibility [88]. Species shift is often constrained by topography, hydrology, soil conditions, and anthropogenic disturbances [89]. For instance, although the geometric centers indicate a northwestward trend, deserts or mountainous regions may hinder the actual dispersal of *A. venetum*, leading to discrepancies between modeled centroid shifts and real-world shifts [90]. Furthermore, this study assumes that soil and topographic factors remain constant under future climate scenarios, introducing additional uncertainty to the predicted distribution patterns.

Nonetheless, the projected centroid shifts reflect macro-scale trends in potentially suitable habitats under climate change. Consistent with this, warming temperatures have been shown to drive upward and poleward shifts in plant species: Lenoir et al. (1986–2005) reported that many alpine species in the Alps migrated to higher elevations, and Zu et al. observed similar altitudinal shifts for seed plants in Gongga Mountain since the mid-1980s [91,92]. These findings highlight climate warming as a major driver of latitudinal and altitudinal shifts in species distributions. Future studies should integrate landscape barriers, species dispersal capacities, and land-use changes to construct more ecologically realistic shift models, thereby improving the accuracy of predictions for species responses to climate change [93].

### 4.4. Resource Conservation and Sustainable Harvesting Strategies

Global climate change poses severe challenges to species survival and ecosystem stability [84]. The survival of a species depends on its ability to migrate to suitable habitats or adapt to unpredictable future climate changes. In this study, we predict that under future climate conditions, the suitable habitat of *A. venetum* will exhibit a trend of northward contraction in certain regions (Figure 6). Due to the extensive harvesting of *A. venetum* roots, rhizomes, bulbs, or entire plants, its demand may significantly exceed supply [94]. Moreover, overharvesting and resource scarcity will accelerate the decline of wild populations, putting them at risk of extinction in regions such as the Xinjiang Uygur Autonomous Region and Inner Mongolia [24]. Conventional in situ conservation measures may be insufficient to prevent the decline of *A. venetum* populations, as habitat destruction caused by human activities has significantly weakened their genetic adaptation potential, making it difficult for them to naturally disperse and adjust their distribution in response to climate change [95]. Against this backdrop, assisted shift has been proposed as a potential conservation strategy, involving the introduction of species into new habitats that are expected to be more suitable for their survival under future climate conditions [96]. Therefore, assisted shift may provide a crucial pathway for the conservation of *A. venetum* germplasm resources. First, systematically collect germplasm resources from natural populations of *A. venetum* across different regions, particularly those at high risk of extinction, such as northwestern Xinjiang, central Inner Mongolia, and central Hebei (Figure 6). Second, introduce *A. venetum* into the future suitable habitats predicted in this study, such as northern Xinjiang, the Hexi Corridor, and central Qinghai, to expand its distribution range (Figure 6 and Figure S1). Third, relocate germplasm resources collected from high-extinction-risk areas to highly suitable habitats, such as eastern Xinjiang, the Hexi Corridor, and northwestern Qinghai, which are predicted to be the optimal habitats for *A. venetum* in the future (Figure 6).

Furthermore, our study predicts that under future climate conditions, the suitable habitat of *A. venetum* will expand toward higher latitudes, while suitable habitats in the Xinjiang Uygur Autonomous Region and Inner Mongolia may degrade or disappear (Figure 6). Therefore, to achieve the sustainable management of *A. venetum*, we propose the following recommendations: (1) Develop and utilize *A. venetum* resources in a sustainable manner, strictly prohibiting illegal harvesting, particularly in high-extinction-risk areas. (2) Collect germplasm resources from threatened populations and introduce them into suitable future habitats. (3) Enhance ex situ conservation of endangered populations through modern biotechnological approaches such as tissue culture. As a perennial plant with both economic and ecological value, *A. venetum* exhibits strong environmental adaptability. Previous studies have shown that due to its broad ecological adaptability, *A. venetum* has been successfully introduced to multiple countries and regions [97]. Additionally, an efficient and stable genetic transformation system has been successfully established [98], laying an important foundation for the conservation and improvement of its germplasm resources.

## 5. Research Prospects

The MaxEnt model is widely favored by researchers due to its stable computational results, high efficiency, and ease of operation [99]. This model can accurately predict the potential distribution of species even with a limited sample size, and it allows for validation of the predictions [100]. However, during the prediction of potential distributions, the MaxEnt model may be prone to overfitting, which can reduce the accuracy of predicting species’ shift capabilities [101]. In order to improve the generalization ability of the model, future research can call the MaxEnt model through the Enmeval optimization package to establish an optimal model, thereby enhancing the prediction accuracy and reducing the risk of overfitting [102].

This study primarily predicts the future suitable habitats of *A. venetum* based on climate variables and environmental factors. However, it does not account for species interactions and human activities, which may affect the accuracy of the predictions. Therefore, future research should integrate multi-factor analyses into species distribution models to improve predictive accuracy and provide a more comprehensive scientific basis for the conservation and sustainable utilization of *A. venetum*. (1) Integration of soil factors to optimize distribution predictions. Current studies mainly predict the future distribution of *A. venetum* based on climate variables. However, soil factors such as soil type, pH, moisture content, and organic matter content also play a crucial role in plant suitability. Future research can enhance the accuracy of predictions by integrating high-resolution soil data into MaxEnt or other species distribution models. (2) Effects of interspecies competition on the suitable habitats of *A. venetum*. Under future climate change scenarios, *A. venetum* may establish new competitive relationships with native or invasive plants, affecting its expansion or survival. Studies can use community ecology models or construct niche competition models to assess the impact of interspecies competition on *A. venetum* distribution, predicting which areas may become new suitable habitats or face competitive exclusion. (3) Assessing the impact of human activities. Human activities, such as land-use changes (agricultural expansion, urbanization), grassland management, and water resource regulation, may significantly impact the survival of *A. venetum*. Future research can incorporate remote sensing data, land-use change models, and policy simulations to analyze how human disturbances affect *A. venetum* distribution and develop conservation and sustainable utilization strategies. (4) Climate adaptation mechanisms and genomic studies. Future research can utilize transcriptome sequencing, metabolomics, and other techniques to investigate the stress resistance mechanisms of *A. venetum* under different climate conditions, identify key drought-, salt-, and cold-tolerance genes, and apply molecular marker technologies to select highly adaptive genotypes for artificial cultivation and germplasm conservation. In summary, future research should build upon spatial distribution predictions while integrating multiple factors such as soil conditions, interspecies competition, and human activities. Additionally, combining molecular biology with ecosystem management will provide a scientific basis for the germplasm innovation, resource conservation, and sustainable utilization of *A. venetum.*

## 6. Conclusions

This study, using the optimized MaxEnt model, integrated five climatic variables, one topographical variable, two soil variables, and two radiation variables to predict the potentially suitable habitats of *A. venetum* across different periods. The AUC values from the model outputs for each period demonstrate that the model accurately simulates the distribution range of *A. venetum*. Among the environmental factors, elevation (Elev), solar radiation in June (Srad6), the mean temperature of coldest quarter (Bio11), and solar radiation in May (Srad5) significantly influence the species’ distribution. Future projections from two GCM models suggest a reduction in suitable habitats, with the distribution center shifting to higher altitudes and latitudes. These findings enhance understanding of the potential distribution patterns of *A. venetum* and provide a scientific basis for defining ecological core areas and conserving genetic resources.

## Figures and Tables

**Table 1:** Description of environmental variables used in MaxEnt.

Category	Variable	Description	Source	Spatial Resolution	Temporal Coverage	Code Unit
Bioclimatic variables	Bio2	Mean diurnal range (Mean of monthly)	WorldClim v2.1	2.5 arc-min	Current and future	°C
	Bio11	Mean temperature of coldest quarter				°C
	Bio15	Precipitation seasonality (coefficient of variation)				mm
Energy variables	Srad5	Solar radiation in May			current	kJ m^−2^ day^−1^
	Srad6	Solar radiation in June				kJ m^−2^ day^−1^
Topographic variable	Elev	Elevation				m
Soil variables	Awc_class	AWC range	HWSD			Code
	T_oc	Topsoil organic carbon				% weight
	T_ph_h2o	Topsoil pH (H_2_O)				−log (H^+^)

**Table 2:** The potential distribution area of the *A. venetum* species in the 2050s and 2090s.

Species	Model	Period	Area of Each Suitable Region (×10^4^ Km^2^)			
			Unsuitable Region	Unchanged Region	Expansion Region	Contraction Region
*A. venetum*	BCC-CSM2-MR	Current vs. SSP126-2050s	703.03	181.31	40.94	14.40
		Current vs. SSP245-2050s	705.34	183.66	38.63	12.05
		Current vs. SSP370-2050s	714.37	169.65	29.60	26.05
		Current vs. SSP585-2050s	704.73	180.64	39.23	15.06
		Current vs. SSP126-2090s	710.44	176.72	33.53	18.99
		Current vs. SSP245-2090s	717.24	176.38	26.72	19.32
		Current vs. SSP370-2090s	702.25	181.26	41.71	14.45
		Current vs. SSP585-2090s	710.20	175.26	33.77	20.44
	MIROC	Current vs. RCP2.6-2050s	692.51	184.63	51.40	11.03
		Current vs. RCP4.5-2050s	688.07	185.88	55.84	9.77
		Current vs. RCP6.0-2050s	685.59	183.88	58.32	11.78
		Current vs. RCP8.5-2050s	708.72	185.61	42.23	13.06
		Current vs. RCP2.6-2090s	684.44	187.23	59.48	8.43
		Current vs. RCP4.5-2090s	673.03	187.90	70.88	7.76
		Current vs. RCP6.0-2090s	690.88	184.08	53.03	11.58
		Current vs. RCP8.5-2090s	684.93	186.33	58.99	9.33

## Data Availability

The original contributions presented in this study are included in the article/Supplementary Material. Further inquiries can be directed to the corresponding author.

## Supplementary Materials

The following supporting information can be downloaded at: https://www.mdpi.com/article/10.3390/plants15060876/s1, Figure S1: Spatial changes in *A. venetum* in China under emission scenarios of the 2050s and 2090s. White, Gray, Red and Blue areas represent not suitable, unchanged suitable, expansion suitable, and contraction suitable areas, respectively. (a–d), the 2050s; (e–h), the 2090s; (a,e), future climate scenario RCP2.6; (b,f), future climate scenario RCP4.5; (c,g), future climate scenario RCP6.0; (d,h), future climate scenario RCP8.5. (Note: general circulation model MIROC5). Figure S2: The bar chart represents the core distribution shift distance for *A. venetum* under different scenarios. Table S1: Model performance of the MaxEnt model calibrated under current climatic conditions. Table S2: Description of environmental variables used in Maxent. Table S3: Contribution of environmental variables to *A. venetum* distribution.
