# Supplementary material for: Conservation—Oriented Analysis of Apocynum venetum’s Distribution in Response to Climate Change Based on MaxEnt Model
Source: Plants (Basel). 2026 Mar 12;15(6):876. doi: 10.3390/plants15060876 (PMC13030657; doi:10.3390/plants15060876)
Supplement: Supplementary file 1 [file plants-15-00876-s001.zip › Table S1.pdf]

Table S1 Model performance of the MaxEnt model calibrated under current climatic conditions

Climatic scenario	AUC values	TSS values
Current	0.966	0.835
