# Supplementary material for: Conservation—Oriented Analysis of Apocynum venetum’s Distribution in Response to Climate Change Based on MaxEnt Model
Source: Plants (Basel). 2026 Mar 12;15(6):876. doi: 10.3390/plants15060876 (PMC13030657; doi:10.3390/plants15060876)
Supplement: Supplementary file 1 [file plants-15-00876-s001.zip › Table S2 .pdf]

Table S2 Description of environmental variables used in Maxent

Code	Description	Unit
bio1	Annual mean temperature	℃
bio2	Mean diurnal range (Mean of monthly (max.temp.-min.temp.)	℃
bio3	Isothermality (bio2 / bio7) (× 100)	%
bio4	Temperature seasonality (standard deviation*100)	℃
bio5	Max temperature of the warmest month	℃
bio6	Min temperature of the coldest month	℃
bio7	Temperature annual range (bio5- bio6)	℃
bio8	Mean temperature of the wettest quarter	℃
bio9	Mean temperature of the driest quarter	℃
bio10	Mean temperature of the warmest quarter	℃
bio11	Mean temperature of the coldest quarter	℃
bio12	Annual precipitation	mm
bio13	Precipitation of the wettest month	mm
bio14	Precipitation of the driest month	mm
bio15	Precipitation seasonality (Coefficient of variation)	mm
bio16	Precipitation of the wettest quarter	mm
bio17	Precipitation of the driest quarter	mm
bio18	Precipitation of the warmest quarter	mm
bio19	Precipitation of coldest quarter	mm
t_ph_h2o	Topsoil pH (H_2O)	−log (H+)
t_cec_soil	Topsoil CEC (soil)	cmol/kg
awc_class	AWC range	Code
t_oc	Topsoil organic carbon	% weight
s_ph_h2o	Substrate-soil pH (H2O)	−log (H+)
s_cec_soil	Substrate-soil CEC (soil)	cmol/kg
s_oc	Substrate-soil organic carbon	% weight
Srad (01 – 12)	solar radiation	kJ m−2 day−1
Elev	Elevation	m
