# Supplementary material for: Conservation—Oriented Analysis of Apocynum venetum’s Distribution in Response to Climate Change Based on MaxEnt Model
Source: Plants (Basel). 2026 Mar 12;15(6):876. doi: 10.3390/plants15060876 (PMC13030657; doi:10.3390/plants15060876)
Supplement: Supplementary file 1 [file plants-15-00876-s001.zip › Table S3.pdf]

Table S3 Contribution of environmental variables to *A. venetum* distribution

Species	Variables	Description	Contribution (%)	Permutation importance (%)	UNITS
	Bio2	Mean diurnal range (Mean of monthly)	4.8	6.2	℃
	Bio11	Mean temperature of coldest quarter	18.1	26.6	℃
	Bio15	Precipitation seasonality (coefficient of variation)	5.5	4.8	mm
	Elev	Elevation	11.5	7.1	m
A. venetum	Awc_class	AWC range	8.5	1.2	Code
	T_ph_h2o	Topsoil pH (H2O)	4.8	0.2	−log (H+)
	T_oc	Topsoil organic carbon	2.1	2	% weight
	Srad5	Solar radiation in May	6.2	3.4	kJ m^{-2} day^{-1}
	Srad6	Solar radiation in June	38.4	48.6	kJ m^{-2} day^{-1}
