# Supplementary figures and images for: Conservation—Oriented Analysis of Apocynum venetum’s Distribution in Response to Climate Change Based on MaxEnt Model
Source: Plants (Basel). 2026 Mar 12;15(6):876. doi: 10.3390/plants15060876 (PMC13030657; doi:10.3390/plants15060876)

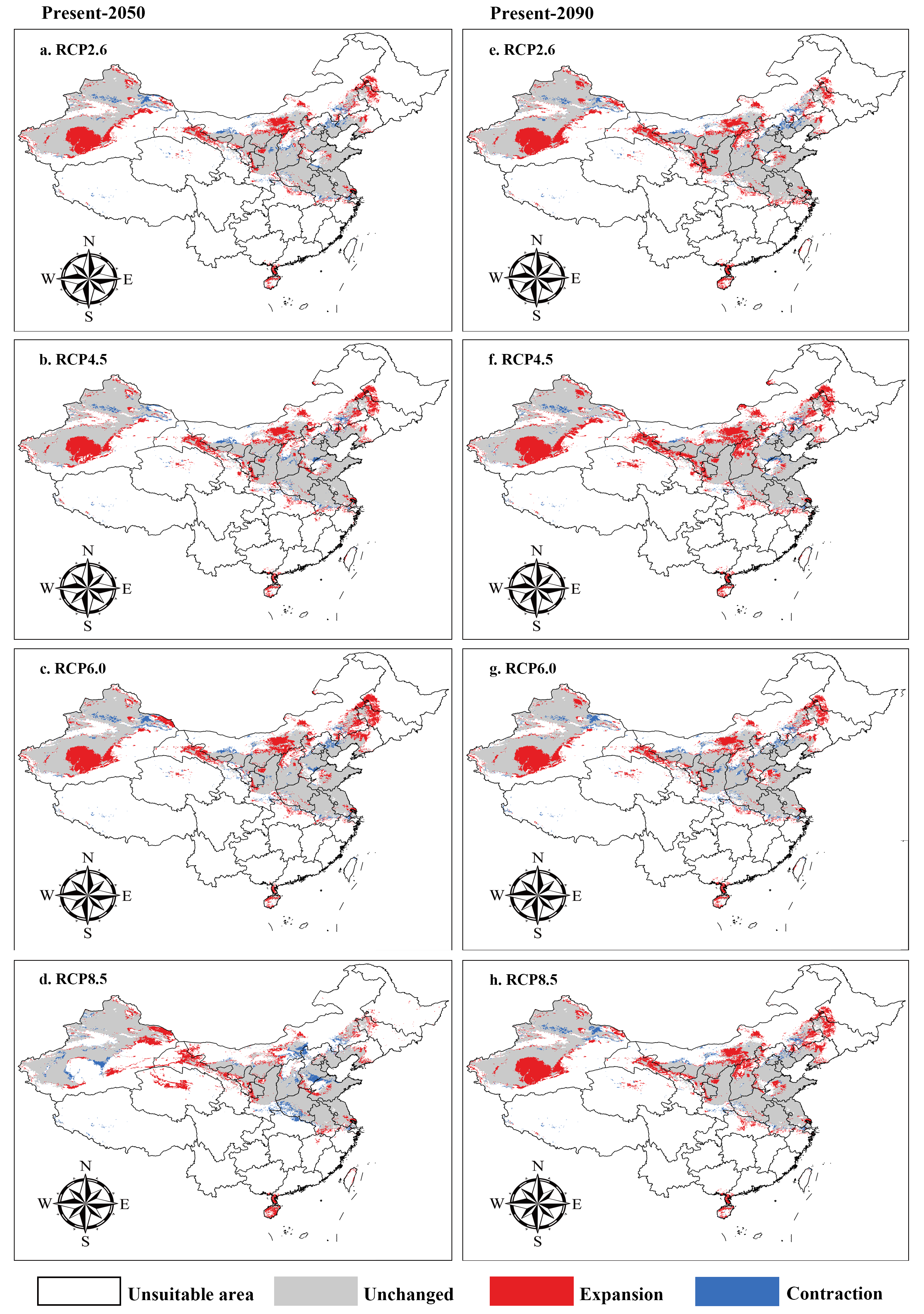

Supplement: Supplementary file 1 [file plants-15-00876-s001.zip › Figure S1.tif]

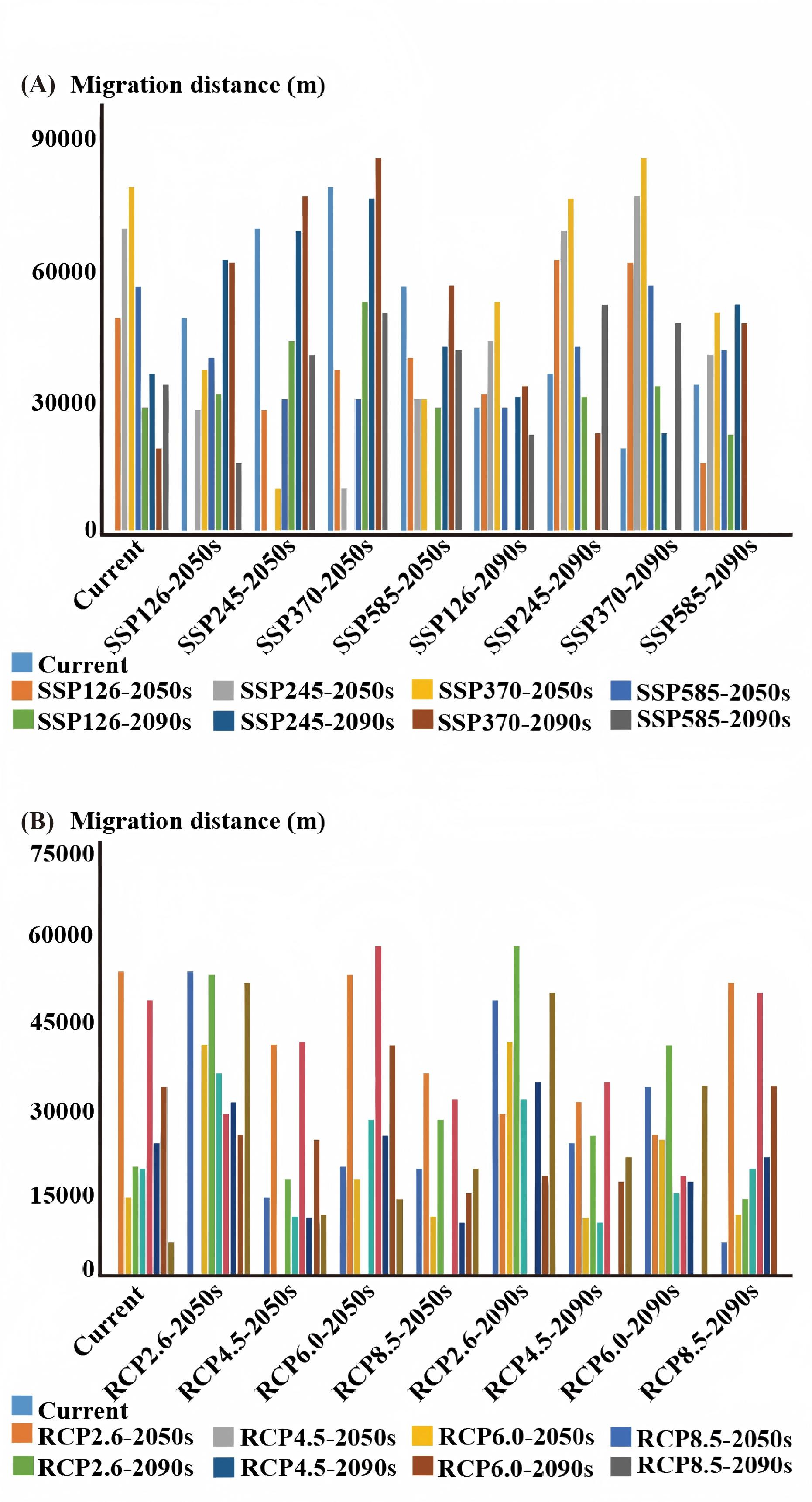

Supplement: Supplementary file 1 [file plants-15-00876-s001.zip › Figure S2.jpg]
